# A Versatile Microarray Platform for Capturing Rare Cells

**DOI:** 10.1038/srep15342

**Published:** 2015-10-23

**Authors:** Falko Brinkmann, Michael Hirtz, Anna Haller, Tobias M. Gorges, Michael J. Vellekoop, Sabine Riethdorf, Volkmar Müller, Klaus Pantel, Harald Fuchs

**Affiliations:** 1 Institute of Nanotechnology (INT) and Karlsruhe Nano Micro Facility (KNMF), Karlsruhe Institute of Technology (KIT), Germany; 2 Physical Institute and Center for Nanotechnology (CeNTech), University of Münster, Germany; 3 Institute of Sensor and Actuator Systems, Vienna University of Technology, Austria; 4 Department of Tumor Biology, University Medical Center Hamburg-Eppendorf, Germany; 5 Institute for Microsensors, -Actuators and –Systems, University of Bremen, Germany; 6 Department of Gynecology, University Medical Center Hamburg-Eppendorf, Germany

## Abstract

Analyses of rare events occurring at extremely low frequencies in body fluids are still challenging. We established a versatile microarray-based platform able to capture single target cells from large background populations. As use case we chose the challenging application of detecting circulating tumor cells (CTCs) – about one cell in a billion normal blood cells. After incubation with an antibody cocktail, targeted cells are extracted on a microarray in a microfluidic chip. The accessibility of our platform allows for subsequent recovery of targets for further analysis. The microarray facilitates exclusion of false positive capture events by co-localization allowing for detection without fluorescent labelling. Analyzing blood samples from cancer patients with our platform reached and partly outreached gold standard performance, demonstrating feasibility for clinical application. Clinical researchers free choice of antibody cocktail without need for altered chip manufacturing or incubation protocol, allows virtual arbitrary targeting of capture species and therefore wide spread applications in biomedical sciences.

The detection and molecular characterization of specific subsets of single cells occurring at extremely low frequencies in body fluids has important potential in biomedicine as diagnostic tool but it is technically still very challenging despite enormous efforts over the past ten years. Fluids such as blood, urine, pleural fluid, cerebral spinal fluid or ascites play a central role in medical diagnostics with the blood being the most widely used source of information. Besides the analysis of cell-derived molecules (e.g., proteins, nucleic acids, and metabolites), the analysis of whole cells circulating in the blood may reveal most complex information about the cause and actual state of a specific disease at the DNA, RNA, and protein level. Examples for applications in basic and applied research are the analysis of rare T-cell subsets in the peripheral blood of patients with immune disorders or infectious diseases^1^ as well as circulating tumor cells (CTCs) in cancer patients which can be regarded as a “liquid biopsy”^2^,^3^, a new diagnostic concept^4^ that has gained enormous interest over the past five years^5^,^6^,^7^,^8^. Distant metastases is the main cause of cancer-related death^9^ and starts with the release of cancer cells from the solid primary tumor (e.g., breast cancer) into the blood stream^10^,^11^. These CTCs can settle into distant organs (e.g., lung, liver, bone or brain) and eventually form metastatic lesions. The analysis of CTCs offers important insights into the biology of metastatic progression and new perspectives in the treatment of cancer metastasis^12^,^13^. However, enrichment and identification of CTCs out of a blood sample is still a major challenge, even after decades of research, as the ratio between CTCs and blood cells is approximately 1:10^9^ (assuming < 200 CTCs/ml, 5 × 10^9^ RBCs/ml, 7 × 10^6^ WBCs/ml)^14^. Many different enrichment strategies for CTCs rely on a positive selection targeting the epithelial cell adhesion molecule (EpCAM) and various microfluidic approaches were developed showing promising results^15^,^16^,^17^,^18^,^19^,^20^,^21^,^22^,^23^,^24^. Anti-EpCAM coated surfaces interact with EpCAM molecules in the cell membrane that immobilize the CTCs, whereas blood cells transmit the system. Verification and further analysis of the captured cells is carried out by immunostaining or other approaches^5^. However, recent studies have shown that EpCAM is not always a reliable marker since also EpCAM-negative CTCs have been discovered in the blood of cancer patients^25^,^26^,^27^. Approaches based on homogeneous antibody coated surfaces struggle with low specificity, making them potentially ineffective for practical applications. On this account, the development of CTC-capturing devices that (i) can easily target a wide range of different surface epitopes, (ii) are able to handle high blood volumes, (iii) exhibit a high specificity, and (iv) allow single cell analysis is still challenging, but highly demanded.

Here, we present a new CTC-capture strategy based on micropatterns that offers high intrinsic specificity, large sample throughput and easy access to captured cells for single cell analysis (Fig. 1) - a streptavidin micropattern on the cm^2^ scale functions as capturing platform for CTCs pre-labeled with biotinylated antibodies. Hence, a large variety of biotin-sensitized cells can be caught by this platform. The micropattern is part of a microfluidic chip that increases the contact probability between labeled CTCs and the micropattern by an integrated herringbone structure^17^. The latter optimizes the flow dynamics to enhance CTC attachments. To demonstrate the clinical feasibility, the micropattern platform was used in clinical samples to isolate CTCs from the blood of breast and colon cancer patients.

## Results

### Design of the microfluidic chip and the integrated micropattern

**Figure 2:**
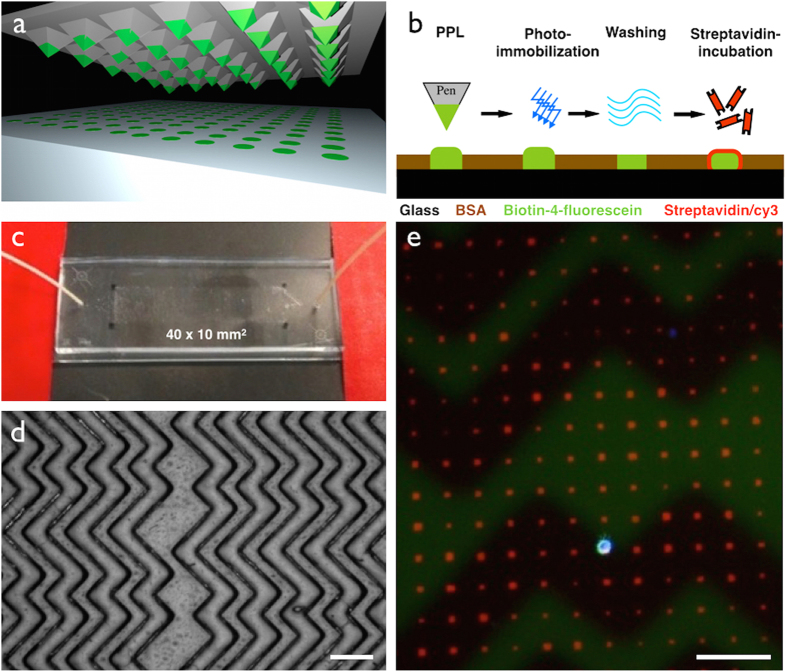
Capturing and immobilization of biotin-labeled CTCs by the micropattern platform. (**a**) The micropattern is fabricated by polymer pen lithography (PPL). The polydimethylsiloxane (PDMS) tips are coated with biotin-4-fluorescein and then precisely navigated onto the surface to generate the micropattern on a 4cm^2^ area. (**b**) This scheme describes the general steps in the generation of the streptavidin array. A microscopy glass slide functionalized with a layer of BSA allows covalent binding of the fluorescein-tag by photobleaching. A topographically flat biotin-array is achieved after washing with PBS. After mounting the fluidic system, the micropattern is further functionalized with streptavidin/cy3. **(c)** A photograph of the microfluidic system fixed on a hot plate. The microarray on the microscopy slide is marked with black dots that correspond to the fluidic chamber. (**d**) The bright field image shows the top channel wall of the microfluidic chip that is responsible for a chaotic stirring stream. The scale bar equals 200 μm. (**e**) This fluorescence micrograph shows a captured cancer cell (DAPI staining, alexa488 secondary staining for anti-EpCAM) trapped on the streptavidin micropattern that is visible in the Texas Red channel. The herringbone structure in the channel ceiling is visible as shadow. The scale bar equals 50 μm.

The capturing strategy presented here is based on a functional dot pattern to enable intrinsic checks for false positive events (c.f. second paragraph in section ‘Identification and single cell extraction’) combined with a microfluidic chaotic mixing system. A standard microscopy slide is coated with bovine serum albumin (BSA) to create both a passivizing and integrative interface: passive, as BSA prevents proteins and cells from unspecific binding; integrative, as fluorescein can bind covalently to BSA molecules induced by photobleaching^28^,^29^. The latter is exploited to generate a chemisorbed dot pattern on the microscopy slide that is stable under streaming liquid condition. Lithography is carried out by polymer pen lithography (PPL), a high throughput technique with full pattern flexibility^30^,^31^. Figure 2a presents a schematic overview of the PPL process: a two dimensional array of polydimethylsiloxane (PDMS) pyramids is brought in contact with a piece of silicon coated with biotin-4-fluorescein acting as an “ink pad”. Capillary forces cause a wetting of the pens that are then navigated above the BSA coated glass slide. The dot pattern is generated by automatically approaching the stamp onto the surface. The piezo-assisted navigation of the stamp or stage respectively guarantees full pattern flexibility. Repeated contact of the stamp with the surface allows to generate a homogeneously dotted surface covering an area of 1 × 4 cm^2^ within two minutes, i.e. 640,000 features in case of a 25 μm dot spacing. Figure 2b illustrates the following steps to achieve the streptavidin pattern. Bleaching the fluorescein-fluorophore leads to a covalent photochemical binding to the BSA and leaves a topographically flat biotin array after washing^29^. The covalent binding makes the pattern very resistant against further washing steps, shear forces in the microfluidics and interactions with the captured cells.

The microfluidic PDMS chip is casted from a prefabricated mold and covalently bonded to the microscopy slide carrying the microarray after oxygen plasma treatment^32^. The array matches the microfluidic channel design with its herringbone topography in the top wall. The micropattern platform can be stored for months before application since the biotin pattern is stable in air and not prone to degradation as compared to antibody patterns. By connecting the microfluidic chip to a syringe, the chamber is flushed with a solution of streptavidin/cy3, which binds to the biotin-array and turns it into the active capture array for biotin-labeled targets. After flushing with phosphate buffered saline (PBS) to remove unbound protein excess, the device is ready for CTC capture. A photo of the microfluidic device is given in Fig. 2c. In order to increase the cell-surface interaction, which is limited in laminar flow conditions, this device has a custom-made herringbone structure in its entire ceiling (Fig. 2d)^33^. The overlaid fluorescence micrographs in Fig. 2e show the streptavidin/cy3 pattern in red and one captured cell from the breast cancer cell line MCF-7 that has been sensitized with biotinylated EpCAM. The cell is stained with an alexa488-tagged secondary antibody against anti-EpCAM and 4′,6-Diamidin-2-phenylindol (DAPI) for nuclear counterstaining (blue). The herringbone structure appears as a shadow in the green channel.

### Advantages of pre-labeling target cells

To proof the principle, defined numbers of cells from cell culture were sensitized with biotinylated epithelial cell specific antibodies, spiked into a suspension of blood cells or untreated cells, isolated by the micropattern platform and finally detected and quantified. Although using the EpCAM-antibody for the model experiments, this device is open for any other biotinylated target and it is feasible for antibody cocktails.

One advantage of pre-labeling the target cells with an antibody cocktail instead of anchoring one type of antibody onto the surface of the capture device is that the fabricated chip has no limitation concerning the type of cell that is to be captured at the same time, as also described by Dickson and co-workers^10^. In fact, any cell (or other target) that can be sensitized by biotinylated antibodies (or other biotin-bearing specific molecules) in a prior incubation step can be captured with one micropattern platform, allowing for great flexibility in this approach: Only the general biotin conjugate will bind to the streptavidin. The binding mechanism is based solely on the interaction between biotin and streptavidin. Even if the incubation protocol varies and different antibodies are used, the fluidic parameters do not change.

Streptavidin dots on the bottom surface function as binding sites. To ensure a saturated binding, 100,000 cells were incubated in 1 mL of PBS carrying 0.5 μg biotinylated anti-EpCAM antibodies representing approximately 8 × 10^12^ molecules (assuming 45 kDa). Prior incubation experiments have shown that antibody labeling should be carried out in a shaker set to 37 °C for 40 minutes (see experimental section).

Currently, anti-EpCAM is the dominantly used marker for CTC capturing and is therefore also used in this study to ensure comparability to other capturing systems. CTCs derived from a solid tumor express EpCAM as epithelial cells, but they may undergo a transition to a mesenchymal phenotype^26^,^7^. This biological process is known as the epithelial to mesenchymal transition (EMT). To our knowledge, most microfluidic surface-marker-based CTC capturing devices focus on EpCAM^34^. This is a severe drawback as EpCAM negative CTCs are invisible for these systems. By labeling CTCs with other specific surface antibodies, such as the human epidermal growth factor receptor 2 (HER-2) in the case of breast cancer (see section “Isolation of CTCs from patients”), or even with an antibody cocktail, the probability to capture EpCAM negative cells will increase. Our capturing device is compatible for the application of antibody cocktails without any further need for adaption.

### Identification and single cell extraction

**Figure 3:**
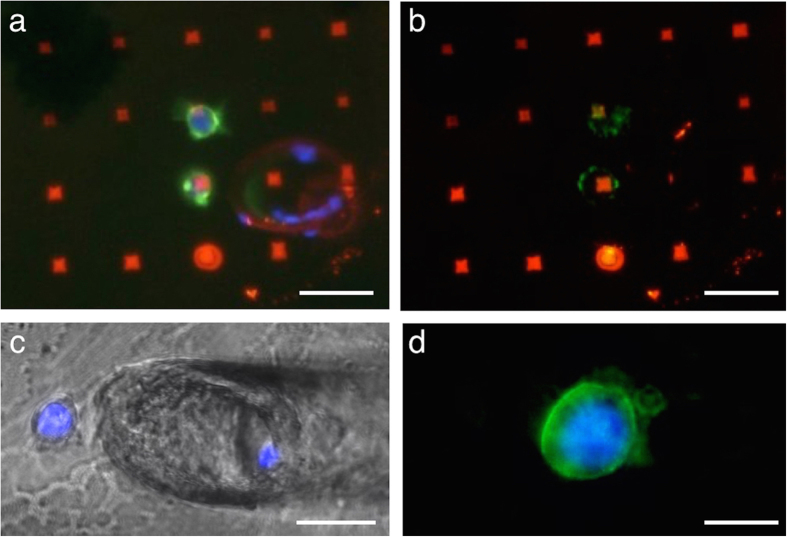
Single cell extraction. (**a**) The micrograph shows an overlay of the Texas Red, FITC and DAPI channel illustrating the streptavidin-pattern in red, the cell nucleus in blue and anti-EpCAM in green of two co-localized MCF-7 cells. A glass micropipette with a diameter of 40 μm is positioned above the cells before extracting them from the pattern. Scale bar equals 30 μm. (**b**) After removal of the cell by micromanipulation, little residues of anti-EpCAM remain on the surface. Scale bar equals 30 μm. (**c**) A capillary deposits two cells on a glass surface that have been picked from a pattern (not corresponding to the cells in (**A**) and (**b**)). Scale bar equals 15 μm. (**d**) The micrograph of the stained cell shows that the cells remain intact after extraction from the pattern. Scale bar equals 10 μm.

Capture approaches based on microfluidic chips aim primarily at CTC quantification, however, immunocytochemistry and fluorescence *in situ* hybridization (FISH) can be conducted on captured cells inside these closed systems. Due to the three-dimensional inner architecture that is homogeneously coated with functional molecules (see CTC- and HB-Chip as well as platforms based on microposts)^17^,^19^,^35^ captured CTCs cannot be further extracted and individually investigated from these systems. It is an advantage to immobilize the cells on a plane surface if further investigation should be performed. The approach presented here is supposed to immobilize CTCs exclusively on the functional micropattern; other surfaces such as the herringbone-structured ceiling and the channel walls are passivated with BSA preventing cells to bind there. Having the captured CTCs on a flat surface allows for picking them by micromanipulation. Figure 3a shows an overlay of fluorescence and bright field micrographs that illustrates the picking procedure. Two trapped cells bind to streptavidin dots; a glass capillary with a radius of 40 μm approaches one cell from the top. The cell is then detached from the dot by putting the aperture over the cell and carefully scratching underneath it. An adjustable inflowing stream loosens the cell and carries it into the capillary. The capillary’s tip can then be navigated into a container and an outgoing stream flushes the cell into it. Figure 3c shows the exemplary deposition of two previously picked cells (not corresponding to the cells in Fig. 3a,b) on a glass surface. Figure 3b shows the same spot as in Fig. 3a after picking the cells from the pattern. The overlay of the Texas Red, FITC and DAPI channel proves that the two cells have been detached from the dots due to the absence of the nucleus stained by DAPI. The dots on that the cells bonded previously have not been damaged. Little green spots around the streptavidin dots remain though. However, we were able to show that cells were still morphologically intact after picking (see Fig. 3d). As the affinity between streptavidin and biotin (being among the strongest in biological systems)^36^ is stronger than EpCAM/anti-EpCAM or the force needed to extract EpCAM from the cell membrane, we conclude that these residues belong to anti-EpCAM with or without attached EpCAM proteins. In principle, the extraction system could be automated, as the captured cells are all positioned on a grid, as defined by the micropattern. Therefore, they can be addressed by the extraction capillary, once an automatic recovery system is registered with the pattern, thus allowing the fully automated recovery of all captured cells.

In contrast to other CTC chips that have a homogeneous antibody coating on the inner walls, our capturing strategy is based on dot microarrays, offering intrinsic specificity enhancement by co-localization detection (Fig. S2). Although all CTC chips are flushed with buffer after the binding procedure, some blood cells will possibly remain by unspecific adhesion. Since the non-CTCs outnumber the actual CTCs by several orders of magnitude, false positive (i.e. non-specifically adhering “captured” cells) are expected to be a serious issue on homogeneously coated surfaces. Although the absolute number of unspecifically adhered cells will not be reduced by the micropattern, the unwanted cells can be identified much easier: the co-localization of our approach leads to a direct sorting into positive and negative cells. After sample incubation, cells that are located on the fluorescently labeled streptavidin dots are expected to carry the biotin anchor and are therefore positive for EpCAM or other corresponding biotinylated antibodies. In contrast, truly negative events, i.e. cells that are located in between streptavidin dots, can be discarded for further analysis, as their chance to be false negative is low. Thus, knowing the position of the dots can verify the capture of a CTC, by performing a simple co-localization analysis between an adhered cell and the capture dot array. In this study, both antibody positive and negative cells remaining on the microarray were stained to clearly evaluate values for efficiency and specificity.

**Figure 4:**
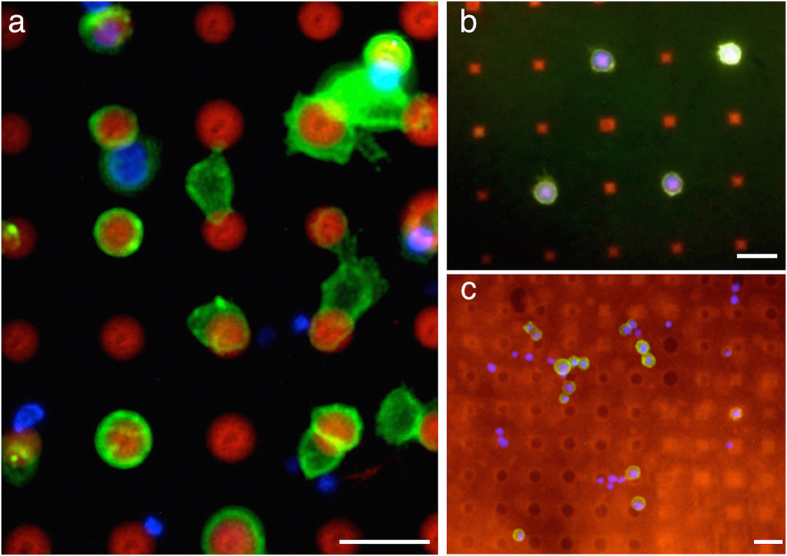
Capturing of sensitized cells by microarrays compared to homogeneous coating. (**a**) MCF-7 breast cancer cells labeled with biotinylated anti-EpCAM (green) are mixed with leukocytes (DAPI staining blue) and incubated on the streptavidin pattern (red). Whereas the positive cells show a clear attraction to the dots, the biotin-negative leukocytes do not show a clear reaction to the pattern. The dot spacing of 20 μm is small enough to allow single cells to bridge dots. By increasing the dot spacing in **(b)**, the individual tumor cells captured on the dots are separated from each other. **(c)** A homogeneous antibody coating does not allow a conclusion about the binding cells solely by the position. The micrograph shows biotin-negative cells (DAPI staining only) and biotin-positive cells (secondary staining in green) randomly binding onto the surface. Cell identification cannot be carried out by the position. Scale bars equal 20 μm in all images.

Figure 4a shows labeled cells from the breast cancer cell line MCF-7, which are stained with an anti-mouse secondary fluorophore against EpCAM, in the green channel, and leukocytes (nucleus stained with DAPI blue) on a streptavidin/cy3 dot pattern in red. The dot spacing is 20 μm. The attraction of the green-labeled cells to the pattern is clearly visible. Leukocytes that also stand out by their smaller size do not sit on dots. These cells are negligible for CTC quantification and further analysis. The narrow patterning allows for some cells bridging between two dots. This is a drawback when observing only the bright field micrograph as these cells would be considered as negative events. Even single cells can potentially bridge two dots if the dot spacing is too narrow. To avoid this, the dot pattern is fabricated with a larger pitch (Fig. 4b). Then, cells can be clearly allocated to dots and considered as positive events.

The need of dot patterns as capturing platform becomes clear when observing Fig. 4c, which shows the result of a capturing experiment utilizing a homogeneously coated streptavidin chip. Here, 50% of MCF-7 cells have been labeled with biotinylated EpCAM; the other half has not been labeled to function as negative cells. When pumping and incubating the cells through a homogeneously coated streptavidin chip, both positive and negative cells remain on the chip; even after washing. The nonspecifically bound negative cells do not allow any conclusion about the cell type, unless further fluorescent staining is performed.

### Performance

The low quantity of CTCs in blood is a challenge for all capturing strategies. A blood volume of 5–10 mL needs to be processed to detect CTCs. E.g. the commercial CellSearch® system produces clinically relevant results with blood volumes of 7.5 mL. When facilitating specific surface proteins as CTC marker, all blood cells need to be brought in contact with an interacting surface.

Our first experiments to proof the stability and binding efficiency of the micropatterns have been performed with 100 μL cell suspension on cover slip platforms. We have created EpCAM micropatterns by direct protein deposition and tried to incubate biotinylated EpCAM on a streptavidin pattern^29^. However, both approaches did not show sufficient binding results during a short-term incubation of 15 minutes. By sensitizing targeting cells with biotinylated EpCAM one can take advantage of the strong affinity between streptavidin and biotin. Live-imaging experiments show that 80% of the biotinylated cells are trapped on a streptavidin dot immediately after touching (see SI video 1). However, using cover slips without microfluidic support will not solve the volume problem, as most of the blood cells will not have a chance to touch the functionalized surface. By designing a microfluidic chip with a herringbone structure enveloping the micropattern, the cell-surface interaction and therefore the binding probability is enhanced.

**Figure 5:**
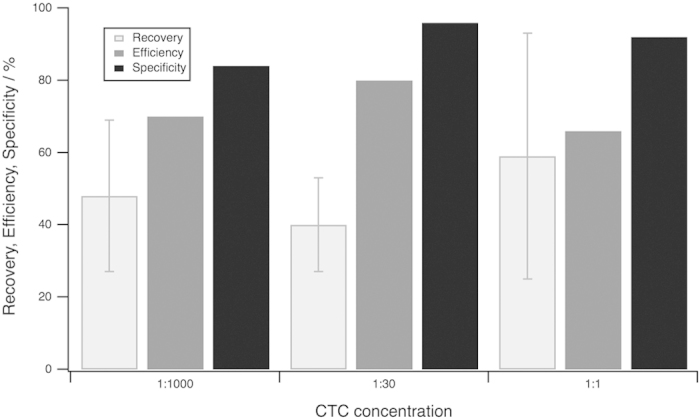
Performance of the presented microarray CTC chip. This diagram faces recovery, efficiency and specificity of three different spiking/dilution experiments. The recovery describes the total performance of the respective experiment. It becomes clear that all three experiments have a similar behavior showing recoveries of 40 to 60%, an efficiency of 60 to 80% and a specificity of more than 85%.

Figure 5 visualizes the homogeneous performance of the chip over different ratios of positive and negative cells. MCF-7 cells have been sensitized with biotinylated EpCAM and were spiked into red blood cell lysed blood carrying 2 × 10^6^ leukocytes or into an untreated cell suspension of MCF-7 cells. These suspensions were subsequently pumped through the micropattern platform while keeping the temperature at 37 °C, as we found that the binding efficiency of biotinylated cells incubated on micropatterns on cover slips was low at room temperature. The binding efficiency decreased about 30% when using the chip at room temperature instead of 37 °C (Fig. S11), as viable cells are more likely to appear at body temperature and these can actively bind to the pattern. Binding effects increase when the cells have a longer contact time to a dot, i.e. less velocity. By interrupting the flow, the cell velocity in the chamber decreases which results in a larger reaction time between cells and dots. Following this protocol, we were able to achieve average recovery rates of about 50% and a high specificity between 86% and 96%.

### Proof-of-principle studies on blood samples from cancer patients

In order to underline the feasibility of the presented system in the clinical settings, a series of blood samples from nine breast and one colon cancer patient was analyzed. Samples from healthy individuals were used as negative controls. We could clearly demonstrate that the antibodies used for the identification of the cells do not show unspecific binding effects, i.e. no keratin signals were seen on blood samples from healthy individuals after FICOLL or Parsortix pre-enrichment (n = 10) (data not shown). Each patient donated a sufficient volume of blood to allow a parallel investigation with the FDA-cleared CellSearch® (7.5 mL of blood per patient) system since this system can be seen as “gold standard” to all other CTC detection methods. Prior to pumping the blood sample through the microfluidic device, CTCs were pre-enriched by size filtration using the Parsortix system (ANGLE Plc) (4 mL of blood per patient) mainly to extract red blood cells and to reduce the number of leukocytes. Cells larger than 10 μm in diameter were captured by the Parsortix system, harvested, and exposed to an antibody cocktail of biotinylated anti-EpCAM and anti-HER2 antibodies. The final pumping step through the micropattern platform was performed following the protocol established for the model system, i.e. at 37 °C and with defined streaming dynamics. Identification of CTCs was realized by immunostaining using the CellSearch® antibody cocktail (Keratin^+^(orange), DAPI^+^(blue), CD45^−^ (red)) for best comparison.

**Figure 6:**
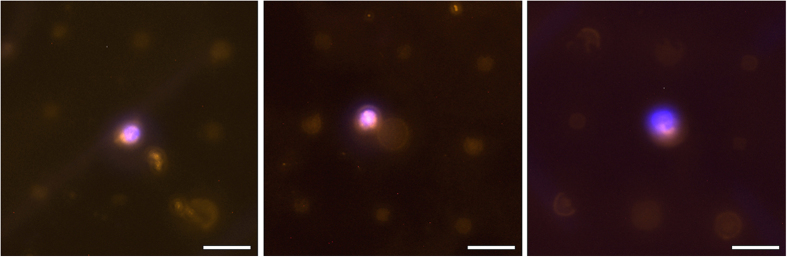
CTCs captured from the blood of a breast cancer patient by micropattern. The fluorescence micrographs show CTCs in contact to the micropattern in different locations on the chip. CTCs are verified by immunostaining using the CellSearch® antibody cocktail. The scale bars equal 20 μm.

CTCs were discovered in 6 out of 10 blood samples using the micropattern platform (range from 0.75–2 CTCs per mL). Four samples were deemed negative since both CellSearch® and the microarray approach were not able to find any CTCs. In 3 of the remaining 6 blood samples CellSearch® found more CTCs than captured by our system (range from 3.7–7.6 CTCs/mL by CellSearch®, 1.25–2 CTCs/mL by the micropattern platform, Fig. S12). However, these patients had high numbers of apoptotic CTCs, which were probably too small to be extracted by the size-based Parsortix system (<10 μm, Fig. S13). Furthermore, the microarray platform is designed for the capturing of viable cells, as we could show that the detection rate decreases for apoptotic ones (Fig. S11). Nevertheless, one capturing experiment indicated the same performance compared to CellSearch®, and two blood samples from patients were even tested positive by our novel device, whereas CellSearch® was negative. This can probably be lead back to the antibody cocktail, which can also capture HER2 positive/EpCAM-negative cells missed by the CellSearch®. Figure 6 shows representative micrographs of CTCs from one breast cancer patient sample captured with the new micropattern platform.

## Discussion

Our strategy for the capture of scarcely occurring cells in large background populations based on a micropattern platform with microfluidic support was demonstrated in the scope of this report with the example of CTC isolation from patient blood. Streptavidin dots function as capturing platform for the target cells pre-labeled with biotinylated antibodies. To maximize the probability of contact between the targets and the micropattern, the platform is encapsulated within a microfluidic PDMS chip, carrying a herringbone structure in its channel top wall that causes a chaotic stirring flow. In contrast to other CTC-chips^5^, the presented approach allows identification of the target cells/CTCs without secondary labeling due to a high specificity and intrinsic additional check against unspecific adhesion by the co-localization with the fluorescent capture dots. Furthermore, it provides the ability to easily pick single target cells by micromanipulation to perform additional molecular and single cell analysis^37^,^38^. Moreover, tumor cells captured by our device are viable, which in principle allows further functional CTC analyses including the establishment of cell cultures and xenografts^6^,^7^,^39^,^40^,^41^ However, the current efficiency of expanding CTCs *in vitro* or *in vivo* is rather low and expansion only works in patients with very advanced disease^7^,^39^,^40^,^41^,^42^. Thus, future studies are needed to focus on this important aspect.

Known as liquid biopsy, frequent quantification and molecular analysis of CTCs before and during treatment could supply a real-time status of the patient, rather than the fixed one-time histopathologic result of the primary tumor^34^. Overt metastases may require many years to develop (e.g., in breast cancer more than 10 years after diagnosis of the primary tumor) and this process is influenced by various selection pressures including cancer treatments such as chemotherapy. Thus, the analysis of the primary tumor–as the current diagnostic approach in clinical oncology-is, therefore, not sufficient to select the best treatment against the metastatic cells emerging from this selection process. Since biopsies of overt metastases is an invasive and difficult procedure depending on the location of the metastatic site (e.g., lung or brain), the analysis of peripheral blood samples for CTCs derived from metastatic sites is an easier alternative.

In the more general approach, free choice of labeling antibody is ensured by the prior incubation or sensitization step, respectively, without further adjustment. Hence, other biotinylated CTC-specific surface proteins can be targeted besides EpCAM. We demonstrated the idea with the application of an EpCAM and HER2 antibody cocktail to include EpCAM-negative CTCs for capture and by choosing altogether different antibodies any other antibody target could be extracted. Hence, one micropattern platform can be facilitated to capture a variety of CTCs, or other cells of interest, which are specifically targeted with antibodies, but generally labeled with biotin.

The presented CTC-chip was demonstrated to be able to capture CTCs from actual patient blood samples, while showing performance comparable or even complementary to the current clinical gold standard (CellSearch®). Achieving this already in this early proof-of-principle study shows the value of this approach for the ongoing development of microfluidic CTC-chips. In contrast to the plethora of microarray platforms developed over the last years^5^,^43^,^44^,^45^, our new device is characterized by a high degree of user-friendly flexibility and the lack of expensive equipment costs. Besides the free selection of antibodies for capture of CTCs (or other cells of interest), our current technology can be combined with almost any pre-enrichment method, including non-biased label-free approaches^5^, as demonstrated in the present investigation. Alternative approaches to capture rare events include flow cytometry which is the most common technique available in most institutions. However, even new generation, high throughput flow cytometers are challenged by the extremely low frequency of CTCs.

Besides CTC analysis future applications might include the analysis of rare T-cell subsets characterized by specific cell surface molecules that can be targeted with antibodies as well as circulating endothelial cells (CECs) and circulating endothelial progenitor cells (EPCs) occurring in pathological processes that disrupt endothelial function, fetal cells from maternal blood, infected cells expressing abnormal surface markers, and bacteria^45^.

## Methods

### Ethic statement

The study was carried out in accordance with the World Medical Association Declaration of Helsinki and the guidelines for experimentation with humans by the Chambers of Physicians of the State of Hamburg (“Hamburger Ärztekammer”). The experimental protocol was approved (Approval No. PVN-3779) by the Ethics Committee of the Chambers of Physicians of the State of Hamburg (“Hamburger Ärztekammer”). All participants gave written informed consent before the study began.

### Micropattern fabrication

The precise protocol for the generation of biotin microarrays by PPL is given in literature^29^ and in the SI in detail.

### Fabrication of the microfluidic device

Device fabrication was accomplished by a rapid, low-cost micromolding technique.

A 4'' glass wafer was used as substrate material for the mold. The inverse of the microfluidic structures were formed within a biocompatible dry film resist (Ordyl SY330, Elga Europe, Italy) with a thickness of 55 μm^46^,^47^. For the mold fabrication, the first resist layer was laminated onto the glass substrate at a temperature of 95 °C, and photolithographically patterned. To achieve the desired herringbone structure in the microfluidic channel top wall, a second layer of dry film resist was laminated onto the structure and patterned, resulting in a total height of 110 μm. After resist development, liquid, degased PDMS was poured over the finished mold. Once solidified, PDMS was peeled off and holes for fluid connections were punched. In a next step, an oxygen plasma treatment of the bottom side of the PDMS was applied. The PDMS was bonded to the micropatterned microscope glass slide. Finally, tubings with syringe needles were fitted into the punched fluid connector holes to finish the microfluidic chip.

### Microfluidics and chip functionalization

The microfluidic chip is connected with 1/32 inch tubes that are inserted into provided inlet and outlet ports. The ingoing tube is connected to a 3-way-cock that is again connected to a syringe. A microfluidic syringe pump (NE-1002X, FisherScientific, USA) is used to reproducibly pump the liquids through the chip. To allow a smooth wetting of the whole chamber and to block unspecific protein binding at the channel walls, 100 μL of bovine serum albumin (1% w/V) and Tween20 (1% V/V) are pumped into the chip and incubated for 30 min. Then, 200 μL streptavidin/cy3 in PBS (0.5% V/V) replaces the prior solution to bind streptavidin to the biotin pattern. The chip is ready-to-use after flushing with 500 μL PBS.

### Blood sampling spiking experiments

4 mL of whole blood from a healthy donor was collected into an EDTA tube. A general density gradient protocol was applied to separate between red blood cells and mononuclear cells: Blood was given in a falcon tube and filled with 30 mL HBSS (Biochrom, Germany) and centrifuged at 400 g and 4 °C for 10 min. The cell pellet was resuspended in 30 mL PBS and 20 mL Ficoll (GE Healthcare, UK) were added. The mixture was centrifuged at 400 g at 4 °C for 30 minutes. The interface and supernatant, containing the mononuclear cells (*i.e.* leukocytes), were transferred to a new 50 ml falcon tube. The tube was filled with PBS and centrifuged at 400 g at 4 °C for 10 min. Supernatant was discarded and cell pellet was resuspended in 1 mL 1 × H-Lysis buffer (R&D Systems, USA) and incubated for 3 minutes with gentle shaking at room temperature. 30 mL PBS was added and sample was centrifuged again at 400 g for 10 minutes at 4 °C. Supernatant was discarded and pellet was resuspended in warm PBS to spike the biotinylated MCF-7 cells into the suspension.

### Blood sampling clinical samples

4 mL (Parsortix in combination with the newly designed microfluidic device) to 7.5 mL of blood (CellSearch®) were drawn from ten cancer patients (breast and colorectal) positive for distant metastases. Blood samples were analyzed for both assays in parallel. The Parsortix system was used as pre-enrichment for CTCs before using the chip assay. For isolation of CTCs using CellSearch®, CTC detection was performed as described elsewhere^48^. The criteria for an event to be defined as a CTC include for both assays: a round to oval morphology, a visible nucleus (DAPI^+^), and a positive staining pattern for an epithelial specific cell (Keratin^+^and CD45^−^).

### Cancer cell sensitization

Cells from the cancer cell line MFC-7 were cultured in Dulbecco’s modified Eagle medium (DMEM) including 10% fetal calf serum (FCS) at 10% CO_2_ in a 25 cm^2^ flask. Cells were washed with warm PBS and detached with trypsin after 2 min incubation and rinsed with warm DMEM. After centrifugation, the cells were re-suspended in warm BSA/PBS 0.1% w/V and counted. 100.000 cells in warm PBS/BSA were then incubated with either 0.5 μg biotinylated Anti-EpCAM (VU-1D9, Abcam, UK) or biotinylated Anti-HER-2 (3B5, Abcam, UK) in a shaker (Eppendorf Thermomixer Comfort, Hamburg, Germany) revolving at 300 rpm at 37 °C for 40 min. The cells are then centrifuged and suspended in warm PBS. The ratio of dead cells is counted before spiking. To achieve a precise number of spiked cancer cells, the suspension is diluted to 10.000 cells/mL; 5 drops of 10 μL suspension are then pipetted on a glass slide and the cells are counted.

### Capturing experiments

Positive and negative cells are mixed in 1 mL PBS and pipetted into a 3 mL syringe (BD Bioscience, USA) that is covered with aluminum foil to hold a temperature of 37 °C. To prevent air bubbles from entering the system, the microfluidic pump is placed vertically (see SI for photos). The pump is set with individual parameters according to the given protocol. A flow rate of 20 μL/min is most commonly used. The chip is placed on a hot plate (Heidolph MR Hei-Tec, Schwabach, Germany) set to 37 °C. The cells leaving the device at the exit are collected in an eppendorf tube. Cells not adhering in the microarray are flushed with 500 μL warm PBS (50 μL/min) and collected in the same eppendorf tube. These cells are then cytospinned on a superfrost glass slide (Menzel-Gläser, Germany) at 2,000 rpm for 3 min, fixed with 4% paraformaldehyde (PFA), stained for the initial EpCAM-label plus DAPI and quantified with a fluorescence microscope. At least five micrographs of low magnification (5×, 10×) of the cytospinned area are taken and average numbers of cells in the whole area are calculated. These numbers are used to obtain the overall recovery. The cells in the chamber can be fixed with PFA for 15 minutes and be stained with DAPI (1:1000) and a secondary antibody against mouse carrying an Alexa488-fluorophore (1:200). To get rid of the excess of staining antibodies, the chip is flushed with another load of PBS (500 μL, 50 μL/min). The number of bound cells is then obtained by counting with an inverted fluorescence microscope. For extracting bound cells with a capillary attached to a micromanipulator, the cells are not fixed. The microfluidic PDMS chip can be cut open with a scalpel to get access to the cells.

## Additional Information

**How to cite this article**: Brinkmann, F. *et al.* A Versatile Microarray Platform for Capturing Rare Cells. *Sci. Rep.* **5**, 15342; doi: 10.1038/srep15342 (2015).

## Supplementary Material

Supporting Information

Supporting Information Video 1

## Figures and Tables

**Figure 1:**
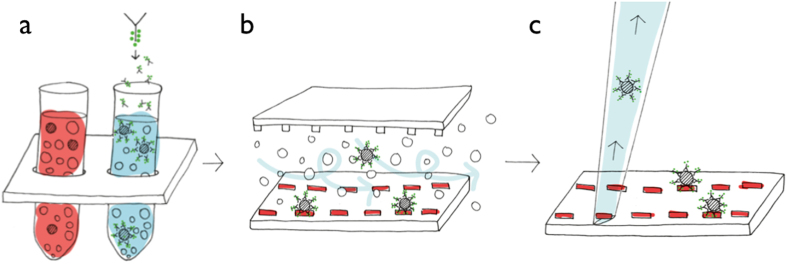
CTC capture and extraction based on the micropattern platform. (**a**) CTCs are incubated with a specific biotinylated antibody, e.g. EpCAM or HER2, or with an antibody cocktail. Red blood cells are eliminated in a prior step by e.g. density gradient protocol or by size-based filtration. (**b**) Pumping the suspension of cells through the microfluidic chip, only the biotin-labeled cells get immobilized on the pattern due to the biotin-streptavidin interaction. A herringbone structure in the chip’s channel ceiling generates a stirring flow that ensures contact events between cells and the micropattern. (**c**) Options for single cell analysis are available after the cells are quantified, optically localized and extracted from the pattern with a micro-capillary. Sample observation can be carried out with totally automated systems (See SI Fig. 7). Artwork reproduced with permission from Jill Enders.
