# Supplementary material for: A Versatile Microarray Platform for Capturing Rare Cells
Source: Sci Rep. 2015 Oct 23;5:15342. doi: 10.1038/srep15342 (PMC4615978; doi:10.1038/srep15342)
Supplement: Supporting Information [file srep15342-s1.pdf]

Supplementary Information for

A Versatile Microarray Platform for Capturing Rare Cells

Falko Brinkmann, Michael Hirtz*, Anna Haller*, Tobias M. Gorges*, Michael J. Vellekoop, Sabine Riethdorf, Volkmar Müller, Klaus Pantel◊, Harald Fuchs◊

Micropattern fabrication by Polymer Pen Lithography (PPL)

A polydimethylsiloxane (ABCR, Karlsruhe, Germany) stamp is molded from a silicon master that was initially generated by photolithography. Oxygen plasma treatment renders the stamp's surface hydrophilic that is needed to homogeneously coat the pyramided tips when approaching the stamp into a stamp pad filled with ink. The stamp pad is a piece of silicon covered with biotin-4-fluorescein ink (Sigma Aldrich, Germany). The stamp is attached to the holder of a NLP2000 system (NanoInk, Skokie, IL, US) that offers a stage able to perform movements over some centimeters with sub-micrometer precision. Depending on the pen's spacing, either several approaches are carried out to realize the pattern with the desired parameters. The stamp itself has an area of 1x1 cm^2. Pen inking is carried out at 70 % relative humidity (RH), lithography at 50 % RH.

A microscopy slide (Menzel Gläser, Germany) acts as substrate for the microarray. Prior cleaning is performed with sonication in chloroform, isopropanol (Merck, Germany) and ultrapure water (18.2 MΩcm) for 10 min each. The slides were then immersed in a solution of bovine serum albumin (BSA, Sigma Aldrich, Germany) and phosphate buffered saline (PBS, Sigma Aldrich, Germany) of a concentration of 3 % w/V. After 45 min, the slides were dipped in PBS 10 times to remove the excess of BSA from the surface and dried with nitrogen. Inking of the PPL stamps was carried out with the stamp pad method as described elsewhere[22]. Cleaned pieces of a silicon wafer were coated with the respective ink mixture by depositing 10-15 µL of ink onto the bare surface by a pipette and allowing the droplet to spread. Then the polymer pens were approached onto this silicon wafer (either by making 10X10 dot patterns with dwell time 1s or by making arbitrary lines) for inking at RH 70 %. Immobilization of the biotin-4-fluorescein micropattern is carried out with a UV lamp (Technotray CU, Heraeus, Germany) at 350 nm for 45 min. The slides are again dipped into PBS 10 times to remove unbound excess ink and dried with nitrogen.

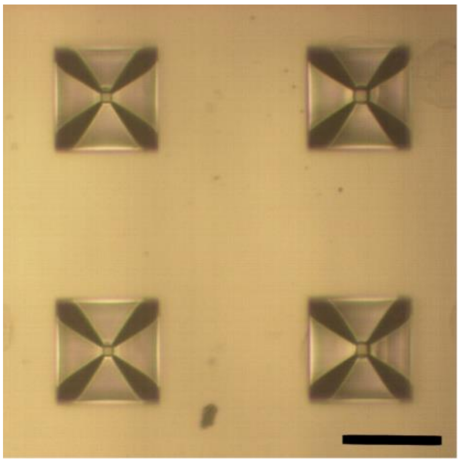

Figure S1. Top view of a PPL stamp during lithography. PDMS pens shaped as pyramids are wetted with biotin-4-fluorescein and brought in contact with a BSA coated microscopy slide to generate the microarray. The squares in the middle of each pen indicate the surface contact area. Scale bar equals 40 um.

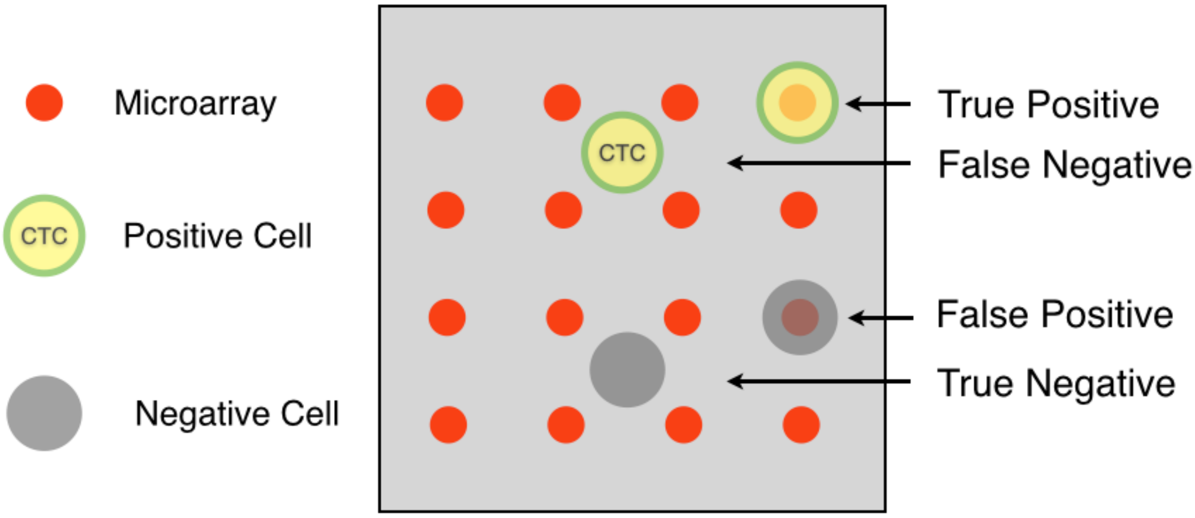

Figure S2. Definition of efficiency and specificity. Distribution of antibody positive and negative cells in the patterned area after a capture experiment. This illustration describes the four possible cases: true positive events (CTC is on a dot), false negative events (CTC is in between dots), false positive events (negative cell is on a dot) and true negative events (negative cell is in between dots). The efficiency is defined as the ratio of true positive events over all CTCs on the array. The specificity is the ratio of true negative cells over all negative cells on the array.

a

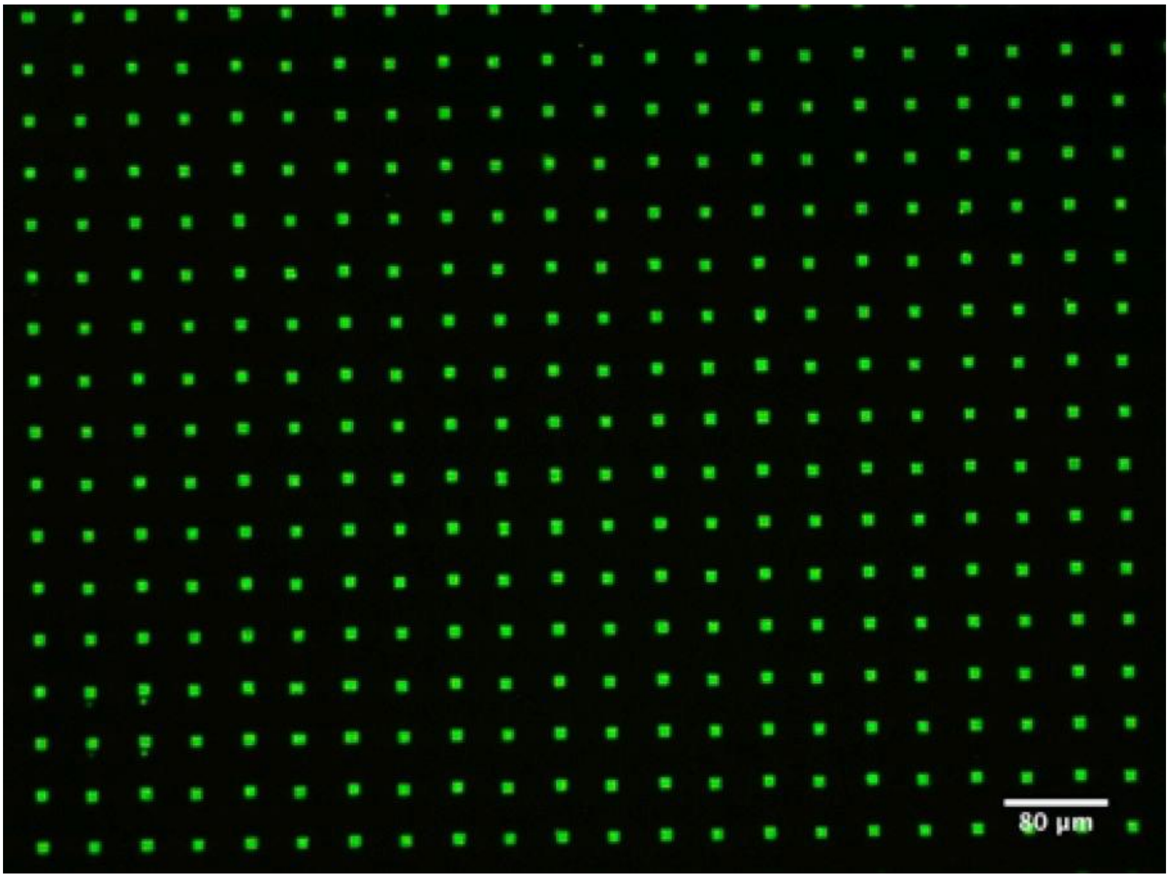

b

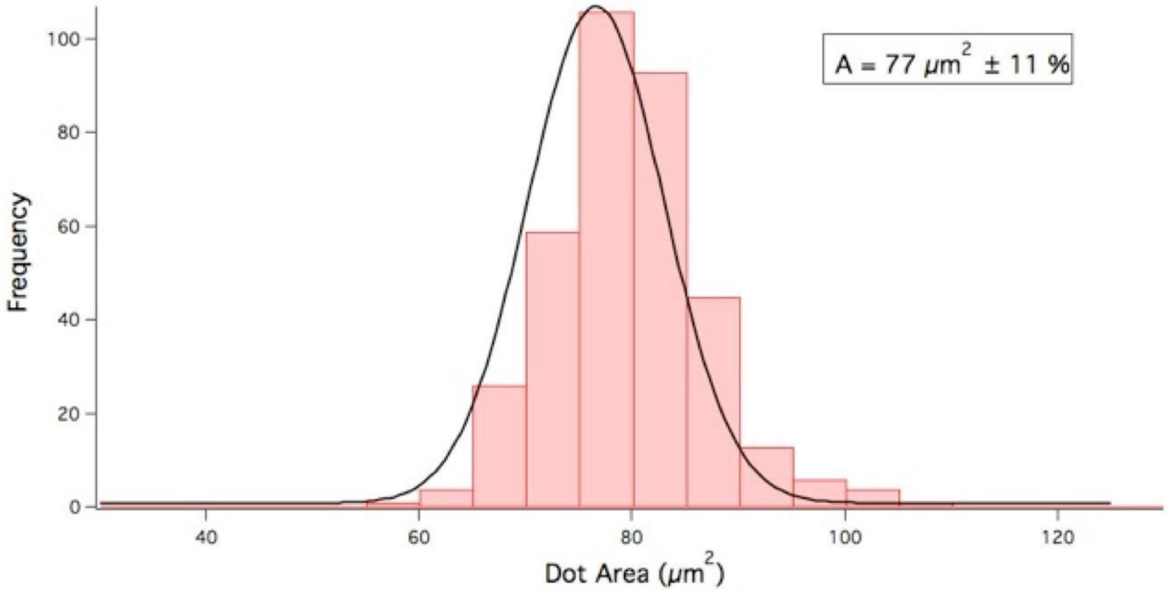

Figure S3. Analysis of the feature size. (a) Fluorescence micrograph of the FITC channel showing a biotin-4-fluorescein array after printing. The size distribution of the feature analysis performed with ImageJ (National Institutes of Health, US) is given in the histogram in (b). The average dot area is 77 $\mu m^2 \pm 11$ % or (8.8 ± 1.0) µm in edge length.

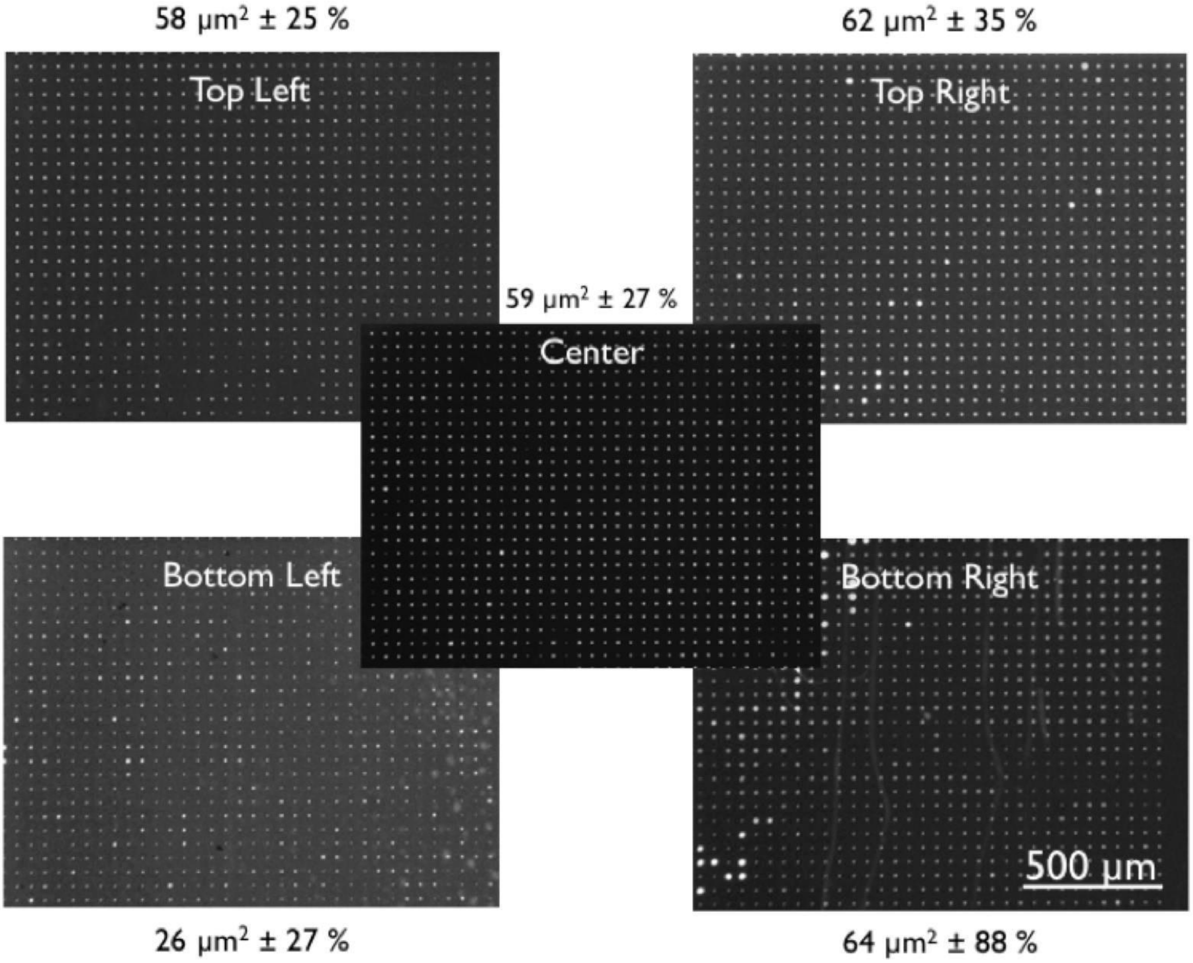

Figure S4. Feature sizes at five different array positions. These fluorescence micrographs were taken of one PPL generated microarray of 1.5 x 1.5 cm^2 area in each corner and in the array center. The pen's spacing is 50 µm. Besides the micrograph in the bottom left of the sample, the average feature size is in between 58 μm^2 and 64 μm^2; indicating that PPL is able to pattern large homogeneous arrays.

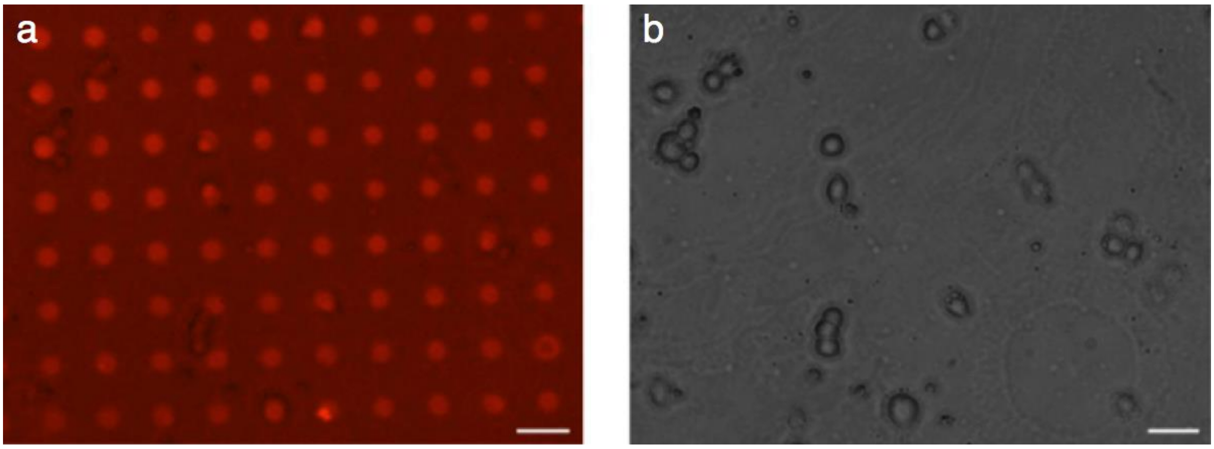

Figure S5. Topographically flat streptavidin-features. (a) The fluorescence micrograph shows a streptavidin\cy3 array in liquid. (b) The brightfield micrograph of the same position with loaded cells. It is not possible to identify the streptavidin pattern. Further investigation of the topographical flat biotin-4-fluorescein pattern on BSA is given in elsewhere[22]. The scale bars equal 20 µm.

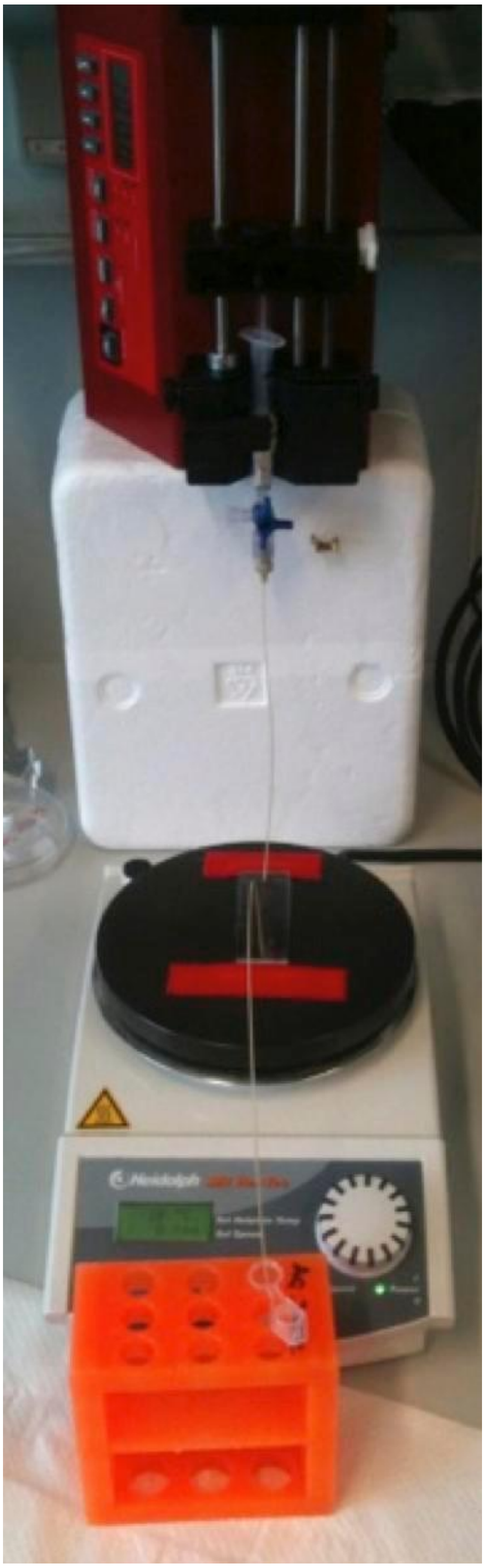

Figure S6. Setup of a CTC capturing experiment with the microfluidic device. A syringe microfluidic pump is positioned vertically to prevent air bubbles from entering the system. A 3-way-cock simplifies the substitution of syringes. A 1/32” tube connects the syringe with the inlet of the microfluidic chip that is placed on a heatable plate. The outlet tube leads into an eppendorf tube to collect the cells flowing out of the chip.

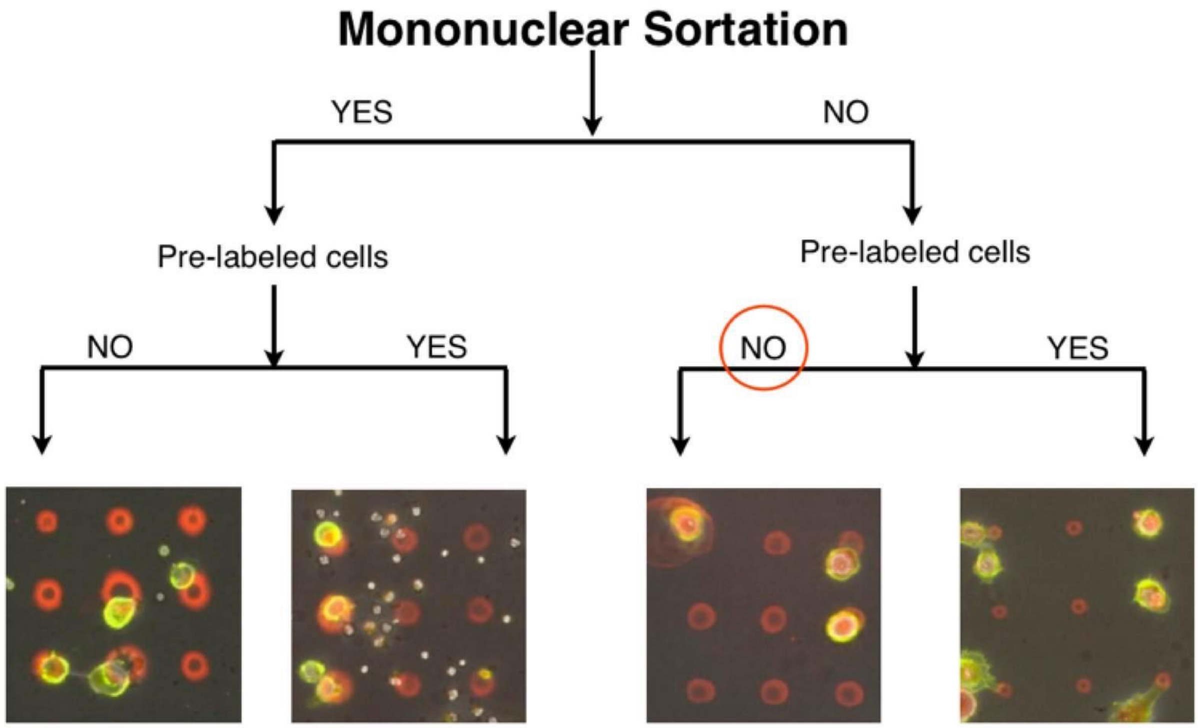

Figure S7. Four different experiments with either whole blood or only leukocytes incubated on a coverslip carrying the microarray. The MCF-7 cells were either labeled before spiking into the blood cell suspension or labeled in the blood cell suspension. The corresponding path to the micrographs is indicated in the diagram. All alternatives show a clear affinity of the labeled cancer cells (secondary antibody staining to Anti-EpCAM) to the dots. The red circle indicated the most desirable way: Cancer cells that are labeled in whole blood and incubated on the microarray with whole blood can be co-localized on the pattern. The dots have proximity of 20 μm each.

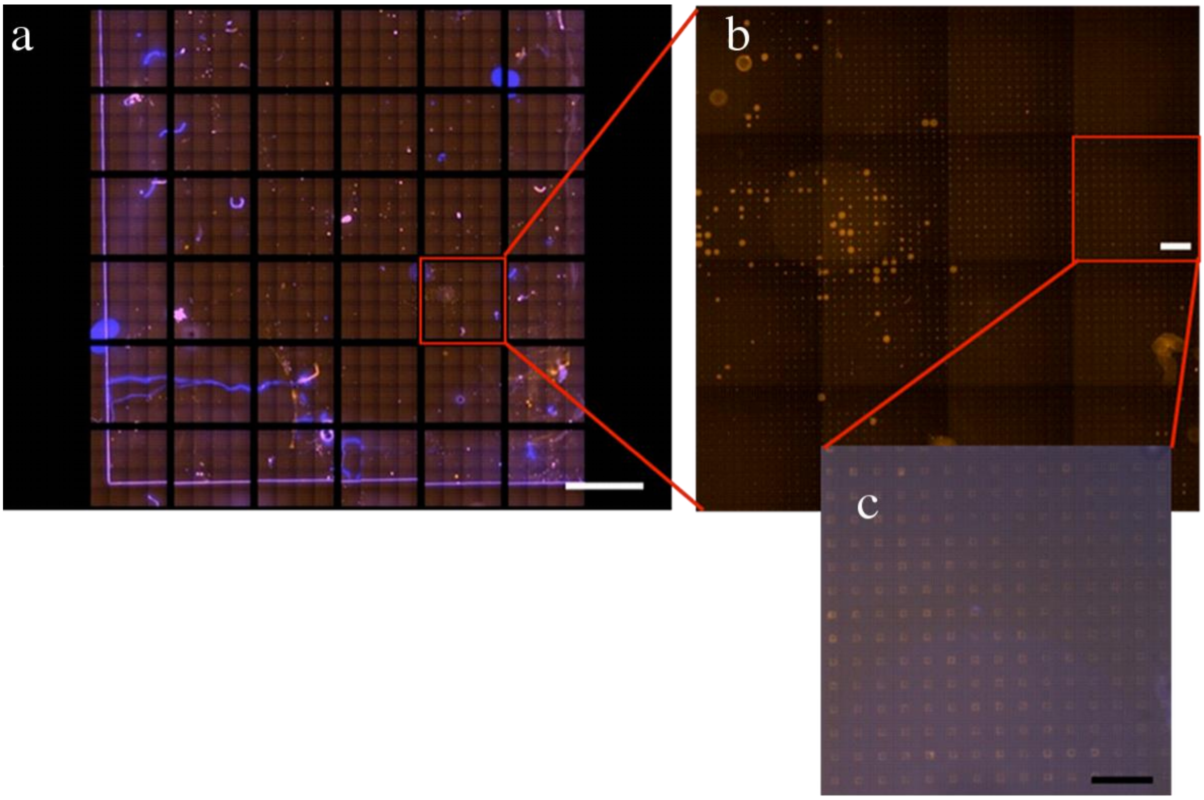

Figure S8. Automated microarray analysis. Cancer cell identification can be performed automatically by using systems like CellaVista (Roche, Switzerland). The chip (here: a cover slip) is placed into the microscope and scanned. As the cells are co-localized on one plane, the cells remain in focus – even over centimeters. This image combines 576 x 2 micrographs of the Texas Red and DAPI channel in one image. Other systems like the CellSelector (ALS, Germany) are able to automatically decide whether a cell is located on a red dot, save the x/y-coordinates and pick the cell from the pattern. Scale bars equal (a) 2.5 mm, (b) 200 µm, (c) 100 µm.

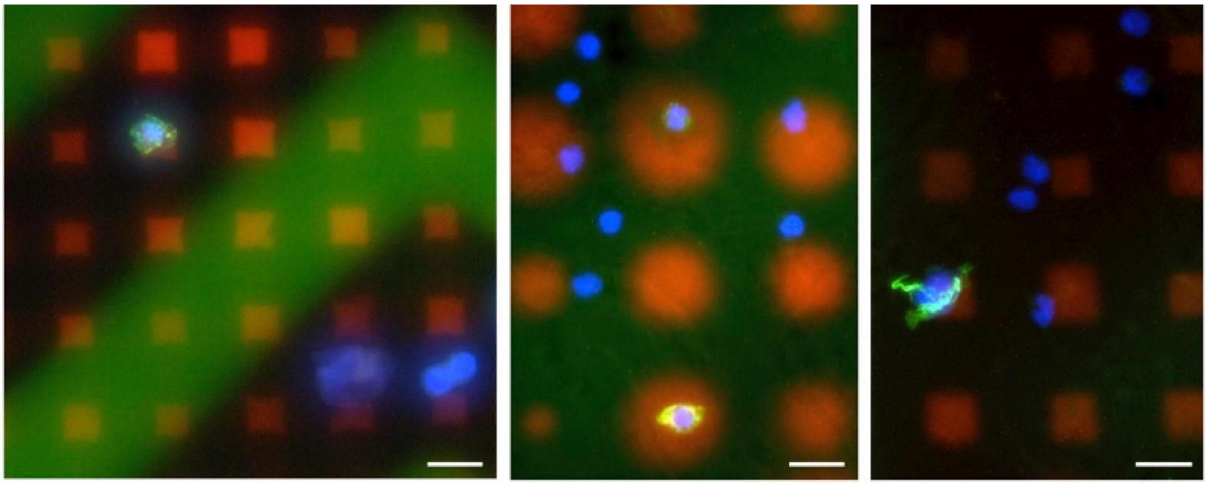

Figure S9. Examples of labeled (green / blue) and untreated (only blue) cancer cells on microarrays using the microfluidic setup. All cells were mixed in 1 mL PBS and pumped through the HB-chip. The scale bars equal 10 µm.

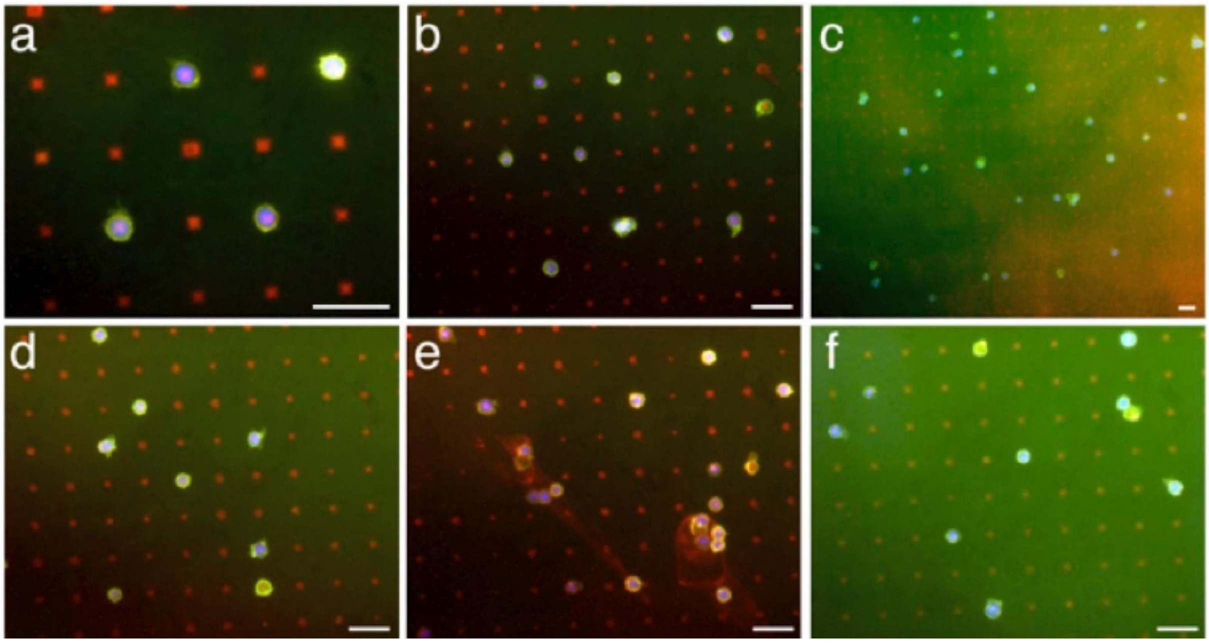

Figure S10. Examples of captured cancer cells (all labeled) on microarrays. 10^6 cells of the cell line MCF-7 were sensitized with 1 µg anti-EpCAM for 40 min. 200 µL suspension was then incubated on a cover slip carrying the streptavidin microarray. The temperature was kept at 37 °C. The scale bars equal 40 µm.

Date	Efficiency	Specificity	Recovery	# of positive cells	# of negative cells	Ratio cells	Used Volume	positive cells transmitting the chip	negative cells transmitting the chip	Flow Rate
**Chip 37 °C**										
**01.09.2013**	**80 %**	96 %	**40 %**	340 ± 55	10000	1:30	1000 µL	**50 %**	20 %	20 µL/min, 1 min, 2 min break
**29.08.2013**	**70 %**	84 %	**48 %**	2050	2x10	1:1000	1000 µL	**32 %**	99 %	20 µL/min, 2 min, 3 min break
**27.08.2013**	**64 %**	92 %	n/a	2000	10000	1:0	900 µL	n/a	n/a	10 µL/min, 3 min, 2 min break, ab 500 durchg.
**26.08.2013**	**66 %**	92 %	**59 %**	2455	2455	1:1	1000 µL	**10 %**	43 %	10 µL/min bis 80 µL, 2 min break (3x) 10 µL/min durchg.
**Chip 25 °C**										
**18.08.2013**	**64 %**	n/a	**13 %**	1000	0	1:0	1000 µL	**80 %**	n/a	10 µL/min, bis 200 µL, 2 min break, durchg.
**15.08.2013**	**54 %**		**31 %**	30000	0	1:0	500 µL	**42 %**	n/a	10 µL/min bis 70 µL,2 min break (4x), dann durchg.
**14.08.2013**	**60 %**	n/a	n/a	6000	0	1:0	1000 µL	n/a	n/a	10 µL/min durchgehend
**Chip cold**										
**05.08.2013**	**25 %**	n/a	**8 %**	450	0	1:0	800 µL	**67 %**	n/a	20 µL/min durchg.
**03.08.2013**	**9 %**		**0,1 %**	70.000	0	1:0	1000 µL	**9 %**	n/a	20 µL/min
**30.05.2013**	**45 %**	1/2	**0,5 %**	10.000	0	1:0	1000 µL	**99 %**	n/a	50 µL/min 50 µL, 2 min break, 50 µL/ min, ...
**Cover Slip**										**Incubation**
**02.05.2013**	64 %	5/8	<1 %	10.000			100 µL			30 min, 37 °C, Shaker after 10 min
**04.05.2013**	32 %	1/3	<1 %	10.000			100 µL			30 min, 37 °C, Shaker 300 rpm always
**04.05.2013**	0 %		0 %	10.000			100 µL			Cold, 10 x 10 µL, 30 min incubation
**08.05.2013**	27 %	2/7	< 1%	96			100 µL			60 min, 37 °C, Shake each 15 min,
**23.05.2013**	32 %	1/3		10.000			in dish			18 hrs in incubator
**20.07.2012**	37 %			570			100 µL			20 min on hot plate without movement

Table S1. Overview of the obtained data after capturing experiments with the microfluidic chip and cover slip incubation. All experiments were performed with cells of the cell line MCF-7 that have been labeled with biotinylated anti-EpCAM in a prior step.

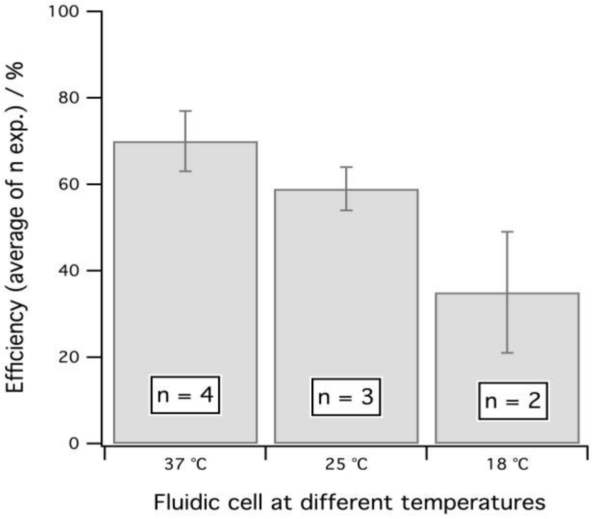

Figure S11. The temperature has a major influence on the chip's performance as the cells lose viability in cold conditions. The average efficiencies of different experiments (SI Table 1) are plotted as a function of the chip's temperature. The efficiency decreases from 70 % to 40 % when lowering the chip's temperature from 37 °C to 18 °C.

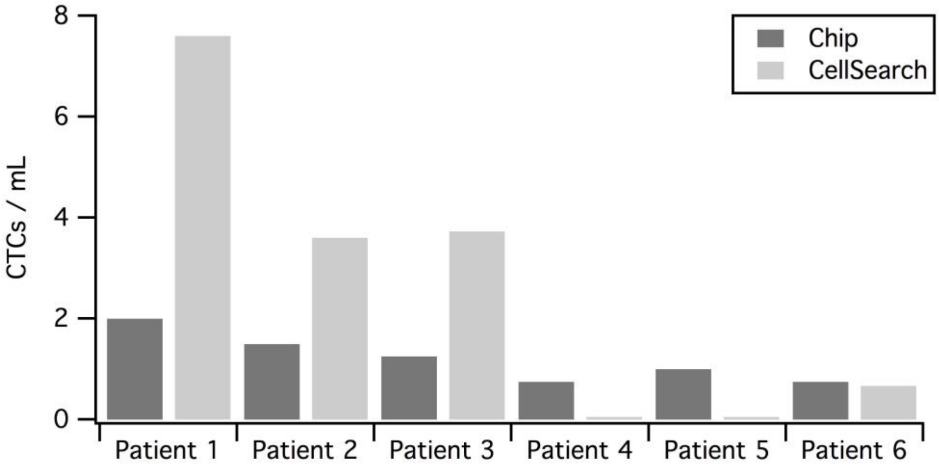

Figure S12. Benchmarking experiment. The bar diagram shows the total number of CTCs per mL detected by CellSearch® and the novel microfluidic device (chip) of different patient's blood samples with positive counts.

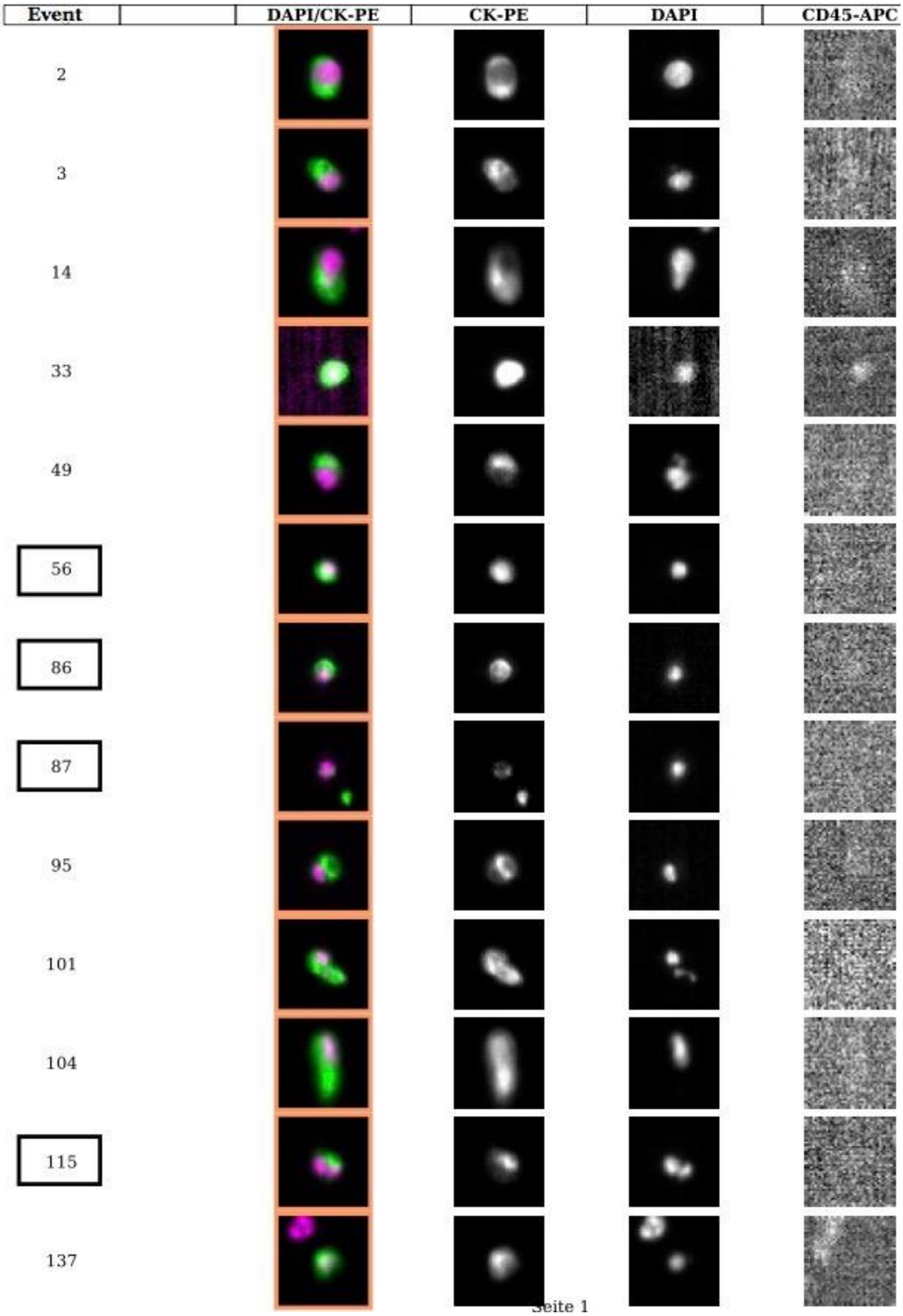
Event
DAPI/CK-PE
CK-PE
DAPI
CD45-APC
2
3
14
33
49
56
86
87
95
101
104
115
137
Seite 1

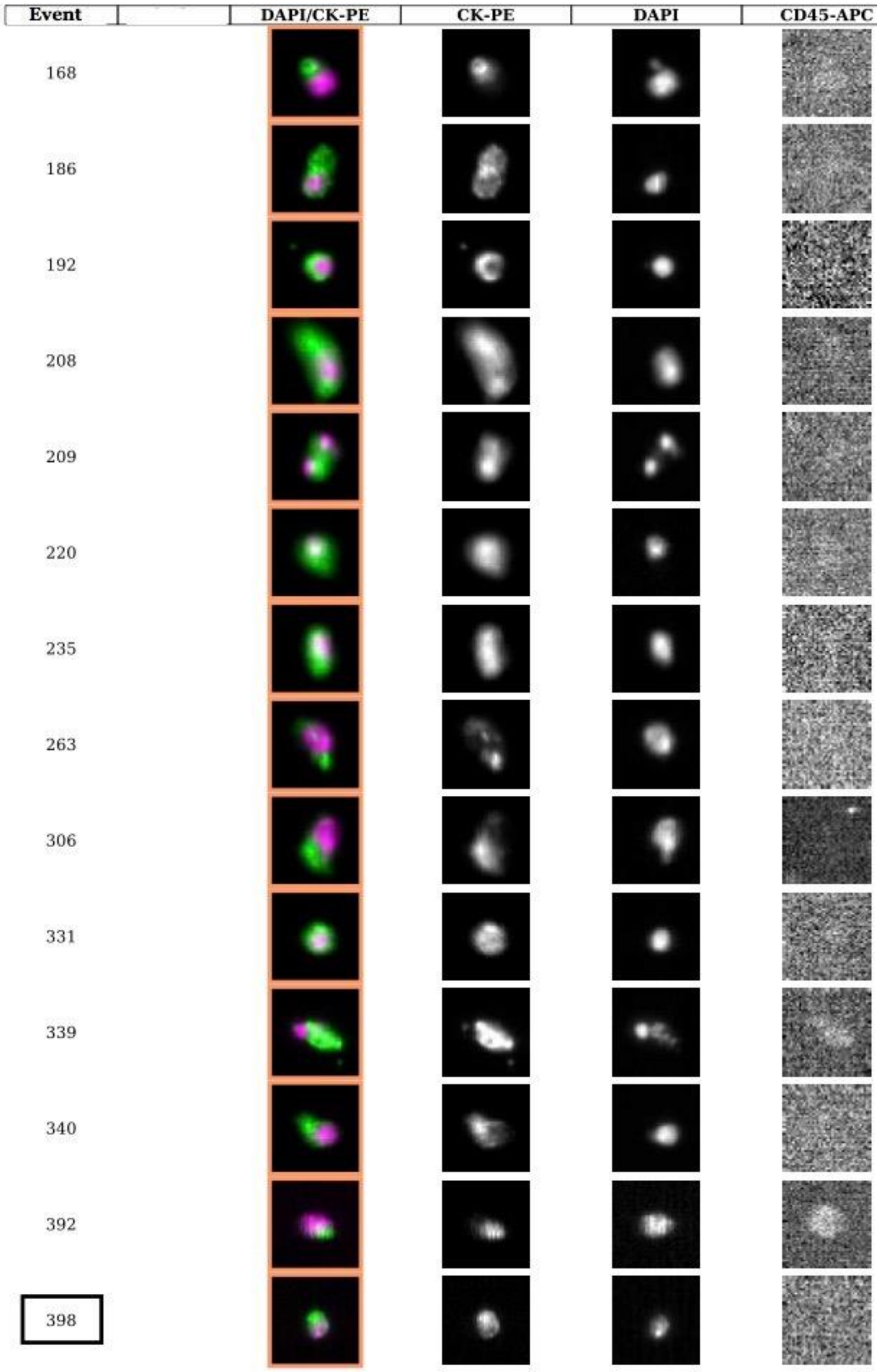

Figure S13. Positive CTC events from blood of a breast cancer patient detected by the Cell-Search® system. Blood from the same sample batch was investigated by the novel micropattern platform in parallel (see Fig. 6, Fig. S12 (patient 2)). Events indicating CTCs too small to be extracted by the Parsortix system, i.e. <10 µm, are boxed on the left.
